# A single center clinical analysis of children with high-risk neuroblastoma

**DOI:** 10.18632/oncotarget.15996

**Published:** 2017-03-07

**Authors:** Xiangdong Tian, Yanna Cao, Jingfu Wang, Jie Yan, Yao Tian, Zhongyuan Li, Huijuan Wang, Xiaofeng Duan, Yan Jin, Qiang Zhao

**Affiliations:** ^1^ Department of Pediatric Oncology, Tianjin Medical University Cancer Institute and Hospital, National Clinical Research Center for Cancer, Key Laboratory of Cancer Prevention and Therapy of Tianjin, Tianjin’s Clinical Research Center for Cancer, Tianjin, People’s Republic of China; ^2^ Department of Radiotherapy, Tianjin Medical University Cancer Institute and Hospital, National Clinical Research Center for Cancer, Key Laboratory of Cancer Prevention and Therapy of Tianjin, Tianjin’s Clinical Research Center for Cancer, Tianjin, People’s Republic of China

**Keywords:** neuroblastoma, high-risk, treatment, surgery extent, survival rate

## Abstract

The current multidisciplinary treatment for patients with high-risk neuroblastoma (NB) is the common census. However, protocols and opinions are different in different regions and institutions. We aimed to assess the protocol formulated by Chinese Children’s Cancer Group study in 2009, and the impact of surgery extent was highlightly evaluated.

**Methods:**

This study enrolled patients with high-risk neuroblastoma between 2009 and 2014 in Department of Pediatric Oncology of Tianjin Medical University Cancer Institute and Hospital. The clinical characteristics of patients were illustrated and surgery extent was evaluated by the impact on survival rate.

**Results:**

The 3-year overall survival (OS) and progression-free survival (PFS) were 56.2% and 50.5%, respectively. LDH (*P*<0.001), bone marrow metastasis at time of diagnosis (*P*=0.001), bone marrow negative after neoadjuvant chemotherapy (*P*<0.001), radiotherapy (*P*<0.001) were significant predictors of OS and PFS. And surgery extent had no impact on the enhancement of high-risk neuroblastoma patients in short time.

**Conclusions:**

This study showed no substantial survival benefit in patients with high-risk NB undergoing gross total tumor resection. Multidisciplinary intensive treatment was essential, especially for patients received subtotal tumor resection. Longer term follow-up is needed to survey complications in surviving patients who received intensive chemotherapy and radiotherapy.

## INTRODUCTION

Neuroblastoma (NB) is the second most common extracranial malignant tumor of childhood that accounts for 7-10% of all pediatric malignancies but is responsible for nearly 15% of children’s cancer deaths [1–3]. The tumor has a preference for young children; 60% of patients are younger than 2 years old and 97% occur before their first decade [4]. During the past three decades, multidisciplinary treatment has been highlighted because more than half of children have widespread metastatic disease at initial diagnosis which increases the difficulty of therapy. Unfortunately, a higher disproportionate percent of children who are older than 1 year have International Neuroblastoma Staging System (INSS) Stage 4 disease, approximately 70% [5–7]. Systemic therapy has led to increasing improvement in treatment of children with the disease and resulted in overall survival rate of 80% or so [8, 9]. But this heterogeneous tumor reflects different degrees of maturation, varying from spontaneous regression or maturity to a quite aggressive and malignant phenotype [10]. For patients with high-risk NB, despite intensive treatment with combination of chemotherapy, surgery, bone marrow transplant, radiotherapy, the outcomes remain poor with long-term survival of less than 50% which would be further decreased by late recurrences and various complications [11–13]. As is reported, relapse at the primary disease site is a major problem of frustrated cases for patients with high-risk NB [14]. Though surgery and radiotherapy play an important role in local control of high-risk NB in most reported protocols, there have been conflicting reports of whether surgery extent improves survival in this cohort of patients [15–18]. A retrospective analysis of patients with high-risk neuroblastoma between 2009 and 2014 attending our institution was performed to demonstrate clinical features and treatment modalities in these patients and to evaluate whether the extent of resection had an impact on survival rate.

## RESULTS

### Patient characteristics

Clinical characteristics of the patients were delineated in Table 1. This study included 52 male patient and 33 female patients (M/F=1.6:1). The median age was 46 months (range: 8–168 months), 14 patients were less than 18 months while 71 aged older than 18 months. Multiple primary lesions occurred in 7 (7/85, 8.3%) patients. Sixty-two lesions (62/85, 72.9%) were in the abdominal or cervix, and sixteen (16/85, 18.8%) in the pelvis or thorax. One patient seemed to have a history of the disease whose two brothers were diagnosed with NB. The majority of patients were in the advanced stage of tumor development (4: n=72 and 3: n=8), according to International Neuroblastoma Staging System (INSS). And there were 4 patients with stage 4s disease, and 1 patient with stage 2A disease. Of the patients with available N-myc status, twenty-five patients (25/85, 29.4%) were N-myc positive, forty-seven patients (47/85, 55.3%) were N-myc negative. Fifty-nine patients (59/85, 69.4%) had serum lactate dehydrogenase (LDH) less than 1500U/L, and twenty-six (26/85, 30.6%) more than 1500U/L. Bone marrow metastasis occurred in 62 (62/85, 72.9%) patients and it turned to be negative after neoadjuvant chemotherapy in 34 (34/62, 54.8%) patients. Thirty-seven (37/85, 43.5%) patients underwent STR and forty-eight (48/85, 56.5%) GTR. Fifty-two patients received radiotherapy (IMRT: n=31, CRT: n=21). Twenty-one patients experienced ASCT, among them, three had only once ASCT.

**Table 1:** Clinical characteristics of patients with high-risk neuroblastoma on CCCG09

Characteristics	Number of patients	%
Sex		
Male	52	61.2
Female	33	38.8
Age		
Median (range)	46 (8-168)	
<18 months	14	16.5
≥18 months	71	83.5
Location		
Abdominal, cervix	62	72.9
Pelvis, thorax	16	18.8
INSS stage		
2A	1	1.2
3	8	9.4
4	72	84.7
4s	4	4.7
N-myc		
amplified	25	29.4
unamplified	47	55.3
unknown	13	15.3
LDH		
<1500U/L	59	69.4
≥1500U/L	26	30.6
Bone marrow metastasis at time of diagnosis		
Positive	62	72.9
Negative	23	27.1
Bone marrow negative after neoadjuvant chemotherapy		
Yes	34	54.8
No	28	45.2
Surgery extent		
STR	37	43.5
GTR	48	56.5
Postoperative complications		
Negative	55	64.7
Positive	30	35.3
Radiotherapy		
IMRT	31	36.5
3D-CRT	21	24.7
No	33	38.8
ASCT		
Yes	21	24.7
No	64	75.3
Therapy response		
CR	34	40.0
VGPR	17	20.0
PR	10	11.8
SD	13	15.3
PD	11	12.9

Abbreviations: CCCG09: Chinese Children’s Cancer Group study 2009; INSS: International Neuroblastoma Staging System; LDH: lactate dehydrogenase; STR: Subtotal resection; GTR: Gross total resection; IMRT: Intensity Modulated Radiation Therapy; 3D-CRT: three dimensional conformal RT; ASCT: autologous hematopoietic stem cell transplantation; CR: complete remission; VGPR: very good partial remission; PR: partial remission; SD: stable disease; PD: progressive disease.

### Treatment outcomes

**Figure 1:**
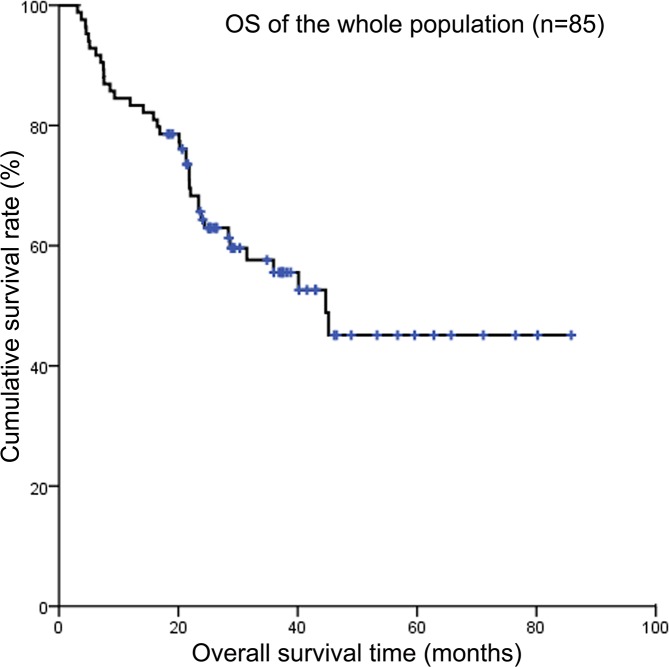
Kaplan-Meier survival curves showing overall survival of the whole population

**Figure 2:**
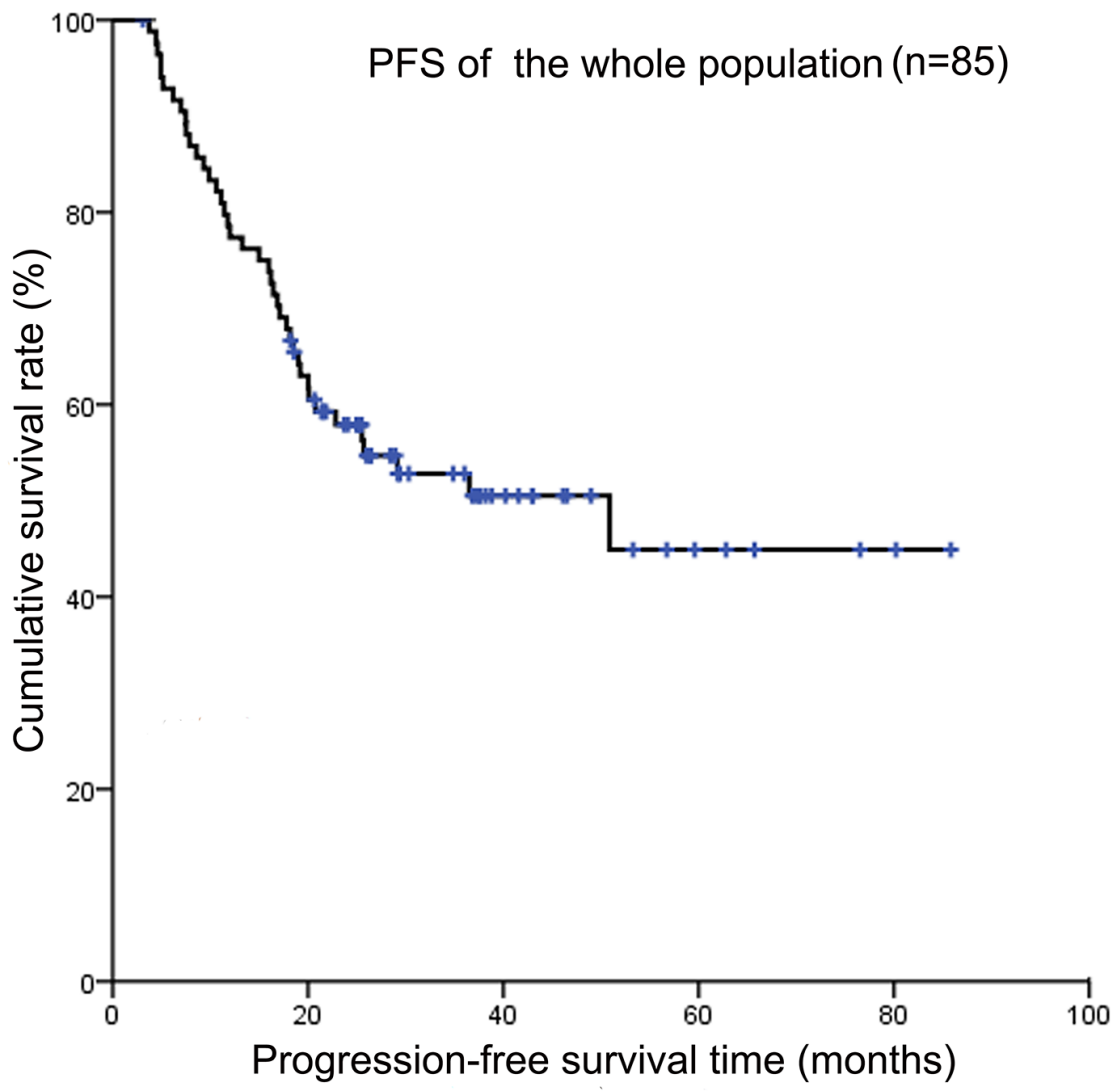
Kaplan-Meier survival curves showing progression-free survival of the whole population

Up to the cut-off date for this analysis, no patient had been lost to follow-up. The 3-year OS rate and PFS rate of the whole population were 56.2% and 50.5%, respectively (Figure 1 and Figure 2). The median survival time (MST) was 25 months (range 3-86 months), 31.8 % surviving 3 years. Forty-three patients were still alive without recurrence and forty-one had died of disease progression or relapse. Although patients experienced therapy-induced complications, nearly all patients could tolerate the intensive treatment. Only one patient died of chemotherapy-induced varicella. Twenty-nine patients relapsed. Among them, recurrent disease was detected at the primary site in only 3 patients, the most common organs of distant relapse involved were bone (n = 13), followed by bone marrow (n = 8), brain (n = 3), and lungs (n = 2). The median progression-free survival (PFS) time was 23 months (range 3-86 months), 27.1 % being progression free at 3 years.

According to univariate analysis (Table 2), LDH (*P*<0.001), bone marrow metastasis at time of diagnosis (*P*=0.001), bone marrow negative after neoadjuvant chemotherapy (*P*<0.001), radiotherapy (*P*<0.001) were significant predictors of OS. Sex (*P*=0.351), age (*P*=0.232), primary location (*P*=0.126), N-myc (*P*=0.066), postoperative complications (*P*=0.218), ASCT (*P*=0.454) were not significantly correlated with OS. Univariate analysis also indicated that primary location (*P*=0.031), LDH (*P*<0.001), bone marrow metastasis at time of diagnosis (*P*=0.002), bone marrow negative after neoadjuvant chemotherapy (*P*<0.001), radiotherapy (*P*=0.002) significantly affected PFS.

**Table 2:** Univariate analysis of clinicopathological features and treatment modalities in the 85 neuroblastoma patients

Characteristics	3-year OS		3-year PFS	
	%	*P*	%	*P*
Sex				
Male	52.4	0.351	48.9	0.21
Female	44.2		37.3	
Age				
<18 months	63.5	0.232	57.0	0.092
≥18 months	47.9		44.4	
Location				
Abdominal, cervix	47.2	0.126	37.1	0.031*
Pelvis, thorax	69.6		67.0	
N-myc				
amplified	42.2	0.066	36.1	0.074
unamplified	51.1		43.7	
LDH				
<1500U/L	70.3	<0.001*	63.3	<0.001*
≥1500U/L	18.7		13.2	
Bone marrow metastasis at time of diagnosis				
Positive	38.2	0.001*	30.0	0.002*
Negative	68.2		65.0	
Bone marrow negative after Neoadjuvant chemotherapy				
Yes	58.7	<0.001*	51.8	<0.001*
No	15.4		11.4	
Surgery extent				
STR	56.0	0.201	48.9	0.087
GTR	42.0		37.3	
Postoperative complications				
Negative	57.3	0.218	47.6	0.107
Positive	39.8		35.8	
Radiotherapy				
Yes	63.2	<0.001*	58.6	0.002*
No	30.7		28	
ASCT				
Yes	65.0	0.454	56.0	0.413
No	56.0		47.7	

* *P*<0.05

Abbreviations: LDH: lactate dehydrogenase; STR: subtotal resection; GTR: gross total resection; ASCT: autologous hematopoietic stem cell transplantation

**Figure 3:**
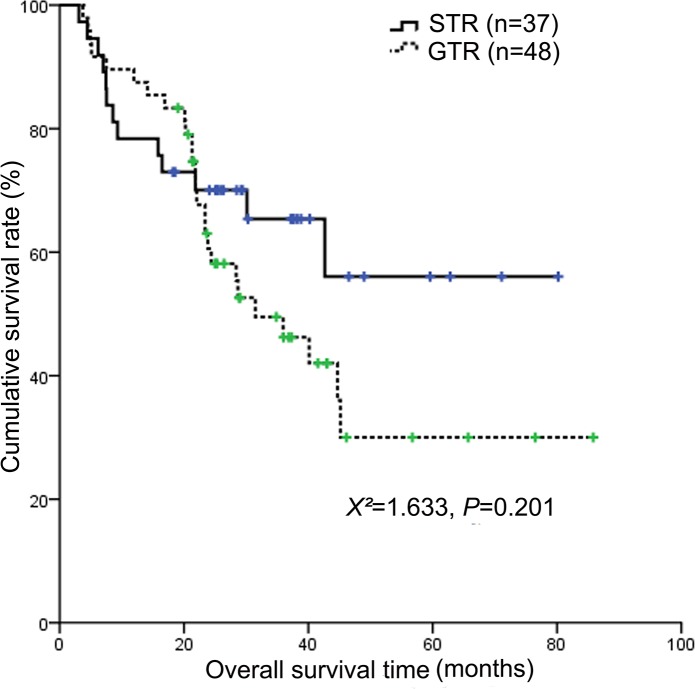
Kaplan-Meier survival curves showing overall survival according to surgery extent Surgery extent did not significantly affect the 3-year OS (*P*=0.201).

**Figure 4:**
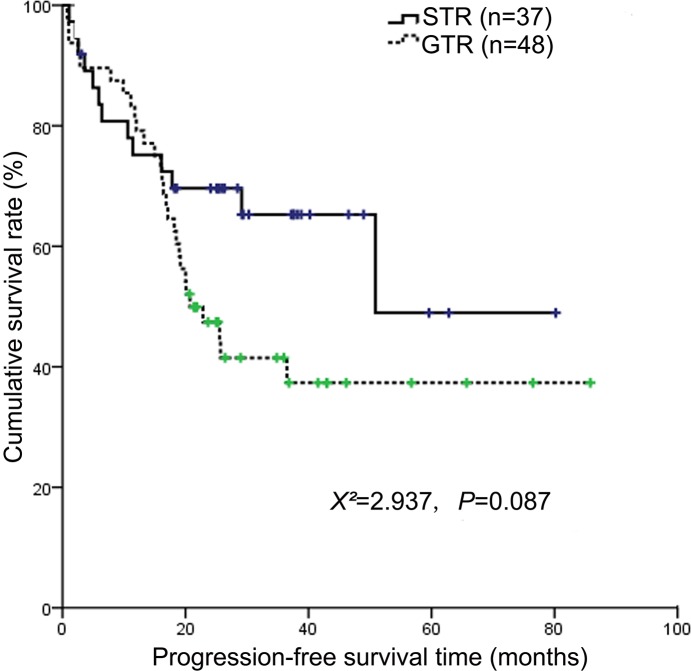
Kaplan-Meier survival curves showing progression-free survival according to surgery extent Surgery extent did not significantly affect the 3-year PFS (*P*=0.087).

Clinical characteristics of patients with NB between two types of surgery were described in Table 3. Surgery extent had no impact on OS and PFS (Figure 3 and Figure 4). And patients underwent GTR faced with more surgery-induced complications than those with STR (*P*=0.006). Furthermore, we analyzed the impact of radiotherapy and ASCT in the subgroup of patients with GTR and STR, respectively. In the subgroup of patients with STR, radiotherapy significantly affected OS (*X*^2^=25.6, *P*<0.001) and PFS (*X*^2^=21.813, *P*<0.001). And in this subgroup, ASCT was significantly correlated with OS (*X*^2^=4.362, *P*=0.037) and PFS (*X*^2^=5.086, *P*=0.024). While in the subgroup of patients with GTR, both radiotherapy and ASCT were not found to have impact on OS and PFS.

**Table 3:** Clinical characteristics of patients with neuroblastoma between two types of surgery

	STR	GTR	*X*^2^	*P*
Age				
<18 months	3^a^	11	3.33	0.083
≥18 months	34	37		
Location				
Abdominal, cervix	27	42	2.886	0.089
Pelvis, thorax	10	6		
N-myc				
amplified	8	17	1.947	0.378
unamplified	23	24		
LDH				
<1500U/L	27	32	0.391	0.532
≥1500U/L	10	16		
Bone marrow metastasis at time of diagnosis				
Positive	25	37	0.959	0.328
Negative	12	11		
Bone marrow negative after neoadjuvant chemotherapy				
Yes	14	15	1.579	0.454
No	12	22		
Postoperative complications				
Negative	30	25	7.693	0.006*
Positive	7	23		
Radiotherapy				
Yes	26	26	2.281	0.131
No	11	22		
ASCT				
Yes	8	13	0.335	0.563
No	29	35		

* *P*<0.05, a: Both groups were doing chi square test for 2 × 2 tables and the Fisher exact ratio are used when expectations are calibrated at time of number less than 5

Abbreviations: LDH: lactate dehydrogenase; STR: subtotal resection; GTR: gross total resection; ASCT: autologous hematopoietic stem cell transplantation.

## DISCUSSION

Patient age at time of diagnosis is an important independent prognostic factor, more than 18 months is associated with greater risk of disease recurrence and lower survival rate [19]. And NB progression and therapy response are often age-dependent [20]. The common use of 18 months as a prognostic factor was unconfirmed in our study. Age did not affect the 3-year OS and EFS rates, probably because of inevitable selected bias that in our study infants in high-risk group were found to be N-myc positive and they could not bear the intensive treatment.

Approximately 65% of NB patients arise in the abdomen, with over half of them in the adrenal gland. Other predilection sites include the neck, chest, and pelvis [1]. Thorax and pelvis are categorized as favorable primary sites, while adrenal, subdiaphragmatic nonadrenal, and cervix are unfavorable [21, 22]. Unconsistent with these reports, we did not find a preferable overall survival rate in thorax and pelvis. It seemed that primary tumor sites were similar in survivors and nonsurvivors. Eventually it did not appear to confer a survival advantage.

Twenty-five to thirty percent of primary NBs are N-myc amplified obtained by fluorescence in situ hybridization (FISH) and N-myc amplification is present in 40% of patients with advanced disease [23]. N-myc amplification is strongly associated with relapse and poor outcome [24–26]. In the present study, N-myc status did not significantly affect the survival rate due to the generally advanced stage and the limited data were insufficient to make firm conclusions.

As a baseline for disease surveillance, serum LDH level at diagnosis correlates to survival rate and is helpful in assessing prognosis [4]. Our survival analysis revealed a statistically significant relationship between OS/PFS and serum LDH level, consistent with the literature. Bone marrow and bone are the most common sites of metastases at diagnosis [4, 23]. In parallel to the literature, 62 patients experienced bone marrow metastasis. Of these, bone marrow metastasis in 34 patients turned to be negative after neoadjuvant chemotherapy. According to the univariate analysis, bone marrow metastasis and its cure time affected the survival rate.

ASCT is considered to have influence on the improved survival rate [27, 28]. While the outcome in our study was not comparable with the previous studies. The nonprospective nature of this study and the small number of patients may have introduced bias. But in the subgroup of patients with STR, ASCT was an important indicator of OS and PFS. Conceivably, for patients with STR, the combined therapy mode was relatively advocated. In the future, more patients would be enrolled in our study to obtain more convincing conclusions about ASCT.

Neuroblastoma is sensitive to radiation therapy, second only to brain tumors [29]. Radiotherapy provides excellent local control in high-risk NB [30, 31], especially IMRT which delivers a conformal dose to the target with minimal damage to normal peripheral issues. The latest protocol for high-risk NB by the Children’s Oncology Group (COG) recommends a dose ranging from 21.6Gy to 36Gy [6]. In the current study, RT did increase the survival rate, especially for the subgroup of patients with STR. To some extent, the effect of STR combined with RT and ASCT was equivalent to that of GTR. Furthermore, observed complications caused by RT were limited and involved only minor toxicities in skin and bowel function. IMRT was feasible, safe in the short run and future clinical trials of high-risk NB should collect data about long-term toxicities about RT.

Treatment of patients with high-risk NB continues to represent a challenge for clinicians and preclinical researchers. Indispensably, surgery in conjunction with RT on primary site improves the local control of high-risk NB, which is a common consensus in most previous studies [32–34]. The contribution of surgery to patients with low-risk and intermediate-risk NB is absolutely apparent and long-term survival rate can reach up to more than 95% with multidisciplinary treatment [32]. But the impact of surgery extent on survival rate for high-risk NBs remains disputable [27, 32–37]. Some researchers argue in favor of gross total resection (GTR) [32–35, 38], while others hold the opposing view [27, 36, 37]. In our study the rate of local failure was 10.3%, much better than that reported in the literature [30, 39, 40]. But no substantial survival benefit was found in the subgroup of patients with GTR. Additionally, the concept of accepting STR to avoid serious complications proved to be successful. The first reason might be the prevailed use of neoadjuvant chemotherapy. Neoadjuvant chemotherapy would not only render extensive, aggressive high-risk tumors more fibrous, less vascular, and more applicable to excision but also eradicate metastasis and offer a clean graft for ASCT. Secondly, effective systemic treatment is often administered in most high-risk NB and the impact of surgery might become smaller with intensive chemotherapy, ASCT and radiotherapy [41]. Though it is impossible to perform a complete resection, the principle of resection at the earliest feasible time should be considered if patients tolerate induction treatment [42]. In the present study, when patients underwent STR, parents were inclined to choose more intensive therapy if economic permitting. And both RT and ASCT did significantly affect the OS and PFS in the cohort of patients with STR. As a result, no impact of surgery extent on the survival rate was found in high-risk NB patients.

In conclusion, our study showed no substantial survival benefit in patients with high-risk NB undergoing gross total tumor resection. For patients received subtotal tumor resection, multidisciplinary intensive treatment was essential. But longer term follow-up is needed to survey complications in surviving patients who received intensive chemotherapy and radiotherapy. Prospective studies are required to evaluate the definite role of surgery in these patients.

## MATERIALS AND METHODS

This study was approved by the Institutional Review Board of Tianjin Medical University Cancer Institute and Hospital, and all guardians of the patients provided written informed consent. The records of Tianjin Medical University Cancer Institute and Hospital from 2009 to 2014 were searched. One hundred and seventy-nine patients with previously untreated NB were reviewed in our institution. Eighty-five of them were confirmed to have high-risk NB and adequate clinical data. Diagnosis was established by two pathologists in our institution based on biopsy of tumors, bone marrow or metastases. Biological factors of the tumor such as histopathologic classification and MYCN were also analyzed. Eligible patients were staged according to International Neuroblastoma Staging System (INSS) criteria and assigned to the high-risk group according to COG risk-stratification scheme [23, 43]. All patients received the majority of their treatment including neoadjuvant chemotherapy, surgery, adjuvant chemotherapy and radiotherapy by the same team of pediatric oncologists. Information regarding patient characteristics, diagnosis and therapy modalities was retrospectively reviewed.

### Treatment

#### Surgery

Primary surgery was performed when the tumor was considered to be resectable at diagnosis. When the tumor was beyond the midline or involved vital nerves or vessels, 4 or 6 courses of neoadjuvant chemotherapy were requisite and patients without disease progression underwent delayed surgery. Gross total resection (GTR) was defined as removal of more than 95% of visible tumor, including regional lymph nodes and surrounding organs infiltrated by tumor. Subtotal resection (STR) was defined as removal of more than 50% but less than 95% of the visible tumor. The extent of surgery was determined by record of the phisician’s operative report and postoperative computed tomography (CT) [38, 43, 44]. Surgical complications were defined as events interfering subsequent treatment included nephrectomy, great vessel/neurological injury, peritumoral organ excision or lesion, tumor rupture, intestinal obstruction, pleural effusion, infection, and hematuria [42].

### Chemotherapy

The chemotherapy regimens mainly consisted of the administration of cyclophosphamide, anthracyclines, platinum and other antineoplastic agents. Patients older than 18 months with stage 4 NB received cyclophosphamide and topotecan, comprising the CI regimen, and cisplatin and etoposide, comprising the PE regimen, and cyclophosphamide, vincristine and adriamycin, comprising the COA regimen (recommended order was CI-CI-PE-COA-PE-COA-CI-PE-COA-CI-PE-COA). Others with high-risk NB turned to an induction backbone of vincristine, cyclophosphamide, cisplatin, etoposide, ifosfamide, doxorubicin and carboplatin (regimen A and B, alternately). Courses were to be repeated every 3 weeks. Eight to twelve courses were recommended. The drugs and dosages prescribed were reported in Table 4. All patients were administered with 160 mg/m^2^ 13-cis-retinoic acid for 14 days per month for half a year, subsequent to the completion of chemotherapy. Chemotherapy-induced severe side-effects mainly included: 1) Grade III-IV myelosuppression after chemotherapy according to the WHO classification; 2) Organ dysfunction required medical treatment, such as hepatic veno occlusive disease (VOD) and cardiac function damage for left ventricular ejection fraction (EF) being less than 60% revealed by cardiac ultrasound examination; 3) Chemotherapy-related agranulocytosis and infection in need of medical care.

**Table 4:** Chemotherapy regimens for high-risk neuroblastoma

Regimen	Drug	Dosage	Days
A	VCR	1.5 mg/m^2^	1,8
	CTX	1000 mg/m^2^	1-2
	DDP	25 mg/m^2^	1-5
	VP-16	100 mg/m^2^	1-5
B	IFO*	1500 mg/m^2^	1-5
	ADR	30 mg/m^2^	1
	CBP	450 mg/m^2^	2
CI	CTX	≥12kg 400 mg/m^2^	1-5
		<12kg 13.3mg/kg	1-5
	TOPO	1200 mg/m^2^	1-5
	or Irinotecan	120 mg/m^2^	1-3
PE	DDP	≥12kg 50 mg/m^2^	1-4
		<12kg 1.66mg/kg	1-4
	VP-16	≥12kg 200 mg/m^2^	1-3
		<12kg 6.67 mg/kg	1-3
COA	CTX*	≥12kg 1800 mg/m^2^	1-2
		<12kg 60 mg/kg	1-2
	VCR	<12 mon 0.017 mg/kg	1-3
		>12 mon and ≥12 kg 0.67 mg/m^2^	1-3
		>12 mon and <12 kg 0.022mg/kg	1-3
	ADR	≥12kg 25 mg/m^2^	1-3
		<12kg 0.83 mg/kg	1-3

* Mesna: 420mg/m^2^, D1-2, q4h*3; Abbreviations: IVgtt, intravenous guttae; VCR, vincristine; CTX, cyclophosphamide; DDP, cisplatin; VP-16, etoposide; IFO, Ifosfamide; ADR, adriamycin; TOPO, topotecan

### Radiotherapy

Radiotherapy was applied after the completion of chemotherapy and surgery. For patients receiving autologous hematopoietic stem cell transplantation (ASCT), radiotherapy was scheduled between the internal of two ASCTs. According to radiologists’ suggestions, patients would receive three dimensional conformal radiotherapy (3D-CRT) or intensity modulated radiation therapy (IMRT) at doses ranging from 16.0 to 36.0 Gy (median, 24.2 Gy) in median single daily fractions of 1.5/1.8 Gy given once daily for 5 days per week. The radiation fields were recommended to encompass the pretreatment primary tumor volume and regional lymph nodes with a 3-cm margin. Metastatic site more than 3-cm also needed radiotherapy [31]. Furthermore, clinical and pathological information was analyzed for the decisions on final dose and clinical target volume (CTV) as well as planning target volume (PTV). Possible complications included myelosuppression, mucositis, skin and soft tissue injury, intestinal discomfort; long-term problems might include skeletal-muscle abnormalization, neurodevelopmental delay, cataracts, deterioration of critical organs and secondary malignancies [24].

### Treatment response

Complete remission (CR): greater than 90% regression of tumor; very good partial remission (VGPR): decrease by 90%-99%; partial remission (PR): more than 50% volume reduction of the primary tumor all measurable metastatic sites; stable disease (SD): no new lesions, < 50% reduction but < 25% increase in any lesion; progressive disease (PD): greater than 25% increase in any preexisting lesion or any new lesion [43].

### Statistical analysis

The statistical software SPSS 22.0 (Chicago, IL, USA) was used to analyze the data. Cumulative survival analysis was performed by the Kaplan–Meier method and the log-rank test was used for single-factor analysis. Overall survival (OS) was calculated from the date of surgery to the time of death from any cause or until the last follow-up. Progression-free survival (PFS) was calculated from the date of surgery to the time of disease progression, recurrence, or death. All *P* values were two-tailed, and *P*<0.05 was considered statistically significant.
